# Formation of Inorganic Sulfate and Volatile Nonsulfated Products from Heterogeneous Hydroxyl Radical Oxidation of 2-Methyltetrol Sulfate Aerosols: Mechanisms and Atmospheric Implications

**DOI:** 10.1021/acs.estlett.4c00451

**Published:** 2024-08-07

**Authors:** Rongshuang Xu, Yuzhi Chen, Sze In Madeleine Ng, Zhenfa Zhang, Avram Gold, Barbara J. Turpin, Andrew P. Ault, Jason D. Surratt, Man Nin Chan

**Affiliations:** † School of Ecology and Applied Meteorology, Nanjing University of Information Science & Technology, Nanjing 210044, China; ‡ Department of Environmental Sciences and Engineering, Gillings School of Global Public Health, The University of North Carolina at Chapel Hill, Chapel Hill, North Carolina 27599, United States; § Atmospheric, Climate, and Earth Sciences, Pacific Northwest National Laboratory, Richland, Washington 99352, United States; ∥ Earth System Science Programme, Faculty of Science, The Chinese University of Hong Kong, and Department of Chemistry, The Hong Kong University of Science and Technology, Hong Kong 999077, China; ⊥ Department of Chemistry, College of Literature, Sciences and the Arts, University of Michigan, Ann Arbor, Michigan 48109, United States; # Department of Chemistry, College of Arts and Sciences, The University of North Carolina at Chapel Hill, Chapel Hill, North Carolina 27599, United States; 7 The Institute of Environment, Energy, and Sustainability, The Chinese University of Hong Kong, Hong Kong 999077, China

**Keywords:** isoprene, secondary organic aerosols, multiphase chemistry, organosulfates, aerosol sulfur cycling, volatile organic products

## Abstract

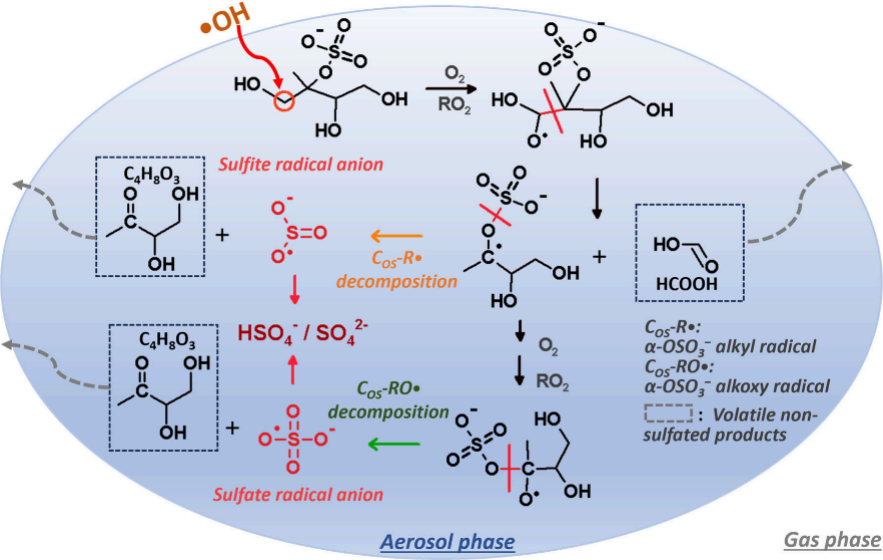

Chemical transformation of 2-methyltetrol sulfates (2-MTS), key isoprene-derived secondary organic aerosol (SOA) constituents, through heterogeneous hydroxyl radical (^•^OH) oxidation can result in the formation of previously unidentified atmospheric organosulfates (OSs). However, detected OSs cannot fully account for the sulfur content released from reacted 2-MTS, indicating the existence of sulfur in forms other than OSs such as inorganic sulfates. This work investigated the formation of inorganic sulfates through heterogeneous ^•^OH oxidation of 2-MTS aerosols. Remarkably, high yields of inorganic sulfates, defined as the moles of inorganic sulfates produced per mole of reacted 2-MTS, were observed in the range from 0.48 ± 0.07 to 0.68 ± 0.07. These could be explained by the production of sulfate (SO_4_^•–^) and sulfite (SO_3_^•–^) radicals through the cleavage of C–O(S) and (C)O–S bonds, followed by aerosol-phase reactions. Additionally, nonsulfated products resulting from bond cleavage were likely volatile and evaporated into the gas phase, as evidenced by the observed aerosol mass loss (≤25%) and concurrent size reduction upon oxidation. This investigation highlights the significant transformation of sulfur from its organic to inorganic forms during the heterogeneous oxidation of 2-MTS aerosols, potentially influencing the physicochemical properties and environmental impacts of isoprene-derived SOAs.

## 1 Introduction

Organosulfates (OSs) play a significant role in contributing to the organic and sulfur content of atmospheric aerosols.^1−3^ Methyltetrol sulfates (MTS), formed through the photochemical oxidation of isoprene under peroxy radical and hydroperoxy radical dominant conditions, are recognized as important and abundant atmospheric OSs, particularly in forested regions with large isoprene emissions under the influence of anthropogenic pollution.^4,5^ Despite comprehensive insights into the formation pathways of MTS,^2−6^ uncertainties with regard to their atmospheric transformation and fate remain. In a prior laboratory study, we investigated the chemical transformation of 2-methyltetrol sulfates (2-MTS, C_5_H_11_O_7_S^–^), the predominant isomers of MTS, through heterogeneous hydroxyl radical (^•^OH) oxidation.^7^ The chemical lifetime of 2-MTS against heterogeneous oxidation was estimated to be ∼16 days under an ambient ^•^OH concentration of 1.5 × 10^6^ molecules cm^–3^. The primary reaction products identified were more oxygenated C_5_ OSs, accompanied by some C_2_, C_3_, and C_4_ OSs in smaller amounts. However, the total sulfur abundance of these detected OSs accounted for <20% of the sulfur released from reacted 2-MTS particles based on their signal intensities during hydrophilic interaction liquid chromatography interfaced with electrospray ionization high-resolution quadrupole time-of-flight mass spectrometry (HILIC/ESI-HR-QTOFMS) analysis in negative ion mode (Section S1 of the Supporting Information). Despite potential uncertainties in the quantification of OSs, it is likely that a significant portion of sulfur from reacted 2-MTS exists in forms other than OSs. Previous studies have identified inorganic sulfates [such as sulfate (SO_4_^2–^) and bisulfate (HSO_4_^–^) ions] as reaction products during the heterogeneous ^•^OH oxidation of other types of OSs.^8−12^ As shown in Scheme S1 (section S2), the formation of inorganic sulfates is attributed to aerosol-phase reactions initiated by sulfate (SO_4_^•–^) radicals originating from the cleavage of C–O(S) bonds upon oxidation. Considering the observed discrepancy in the aerosol sulfur mass balance before and after the heterogeneous ^•^OH oxidation of 2-MTS, it is suggested that the missing sulfur may exist in the form of inorganic sulfates. Additionally, there is speculation that nonsulfated products can be formed alongside inorganic sulfates during the heterogeneous ^•^OH oxidation of 2-MTS. Depending on their volatilities, these products might evaporate into the gas phase, leading to a reduction in the size and mass of aerosols. It is postulated that aerosol mass loss might correlate with the amount of inorganic sulfates formed upon oxidation.

To test these hypotheses, we revisited the heterogeneous ^•^OH oxidation of 2-MTS aerosols using an oxidation flow reactor (OFR) coupled with online and offline analytical methods (Scheme S2). 2-MTS aerosols exiting the reactor were collected by using a particle-into-liquid sampler (PILS) before and after heterogeneous oxidation. The PILS samples underwent analysis through HILIC/ESI-HR-QTOFMS and ion chromatography (IC) to quantify the amounts of 2-MTS and inorganic sulfates, respectively. Variation in aerosol size and mass measurements upon heterogeneous oxidation was measured by using a scanning electrical mobility spectrometer (SEMS). In particular, we examined the significance of the conversion of sulfur from organic to inorganic forms by quantifying the amount of inorganic sulfates formed during the heterogeneous ^•^OH oxidation of particulate 2-MTS. Moreover, we discuss how the sulfur conversion processes affect the size and mass of aerosols upon heterogeneous oxidation. Tentative reaction mechanisms are also proposed in this study to elucidate the formation pathways of inorganic sulfates and nonsulfated products upon oxidation.

## 2 Methods and Materials

Scheme S2 illustrates the experimental setup for the heterogeneous ^•^OH oxidation of 2-MTS aerosols. Detailed experimental procedures can be found in our previous study.^7^ In brief, we synthesized 2-MTS in house following established procedures.^13^ Heterogeneous oxidation experiments were carried out in the OFR at 63.0 ± 0.4% relative humidity (RH) and 22.4 °C. To generate 2-MTS aerosols, we atomized an aqueous solution containing 1.04 mM 2-MTS standard through a nebulizer. Hydrolysis of 2-MTS in the atomizer was not expected on the time scale of our experiments because they are stable for ∼28 days.^14,15^ These aerosols were then mixed with gases [nitrogen (N_2_), oxygen (O_2_), and ozone (O_3_)] before being directed into the reactor. Within the reactor, aerosols underwent heterogeneous oxidation by gas-phase ^•^OH, generated through the photolysis of O_3_ under ultraviolet (UV) light (λ = 254 nm) in the presence of water vapor. The ^•^OH concentration inside the reactor was varied by adjusting the UV light intensity. The ^•^OH exposures, representing the product of the gas-phase ^•^OH concentration and aerosol residence time (128 s), were determined to be in a range from 0 to 19.8 × 10^11^ molecules cm^–3^ based on a modeling method.^7^ This is equivalent to ∼15 days of atmospheric oxidation, assuming an ambient ^•^OH concentration of 1.5 × 10^6^ molecules cm^–3^.^16^ After heterogeneous oxidation, the aerosol flow leaving the reactor passed through a carbon strip denuder (Sunset Lab) and an O_3_ denuder (Carulite 200 Catalyst, Carus) before being sampled by a SEMS for aerosol size and mass measurement and by PILS (BMI) for chemical analysis.

For chemical analysis (Scheme S2), PILS samples were collected at 5 min intervals. A 25 μM solution of lithium bromide (LiBr) served as an internal standard to correct for dilution caused by water vapor condensation during collection. An aliquot of each PILS sample was injected into an Agilent 6500 Series UPLC system equipped with a HILIC column coupled with an electrospray ionization (ESI) source and a quadrupole-time-of-flight mass spectrometer (QTOFMS, Agilent 6250) to quantify the 2-MTS and detect OSs formed upon heterogeneous ^•^OH oxidation. Inorganic sulfates (HSO_4_^–^ and/or SO_4_^2–^) formed upon oxidation were quantified by using an IC system (ICS 3000, Thermo Fisher). Detailed sample preparation and operating conditions for HILIC/ESI-HR-QTOFMS and IC analyses are given in Sections S1 and S3, respectively.

## 3 Results and Discussion

### 3.1 OSs and Inorganic Sulfates

**Figure 1:**
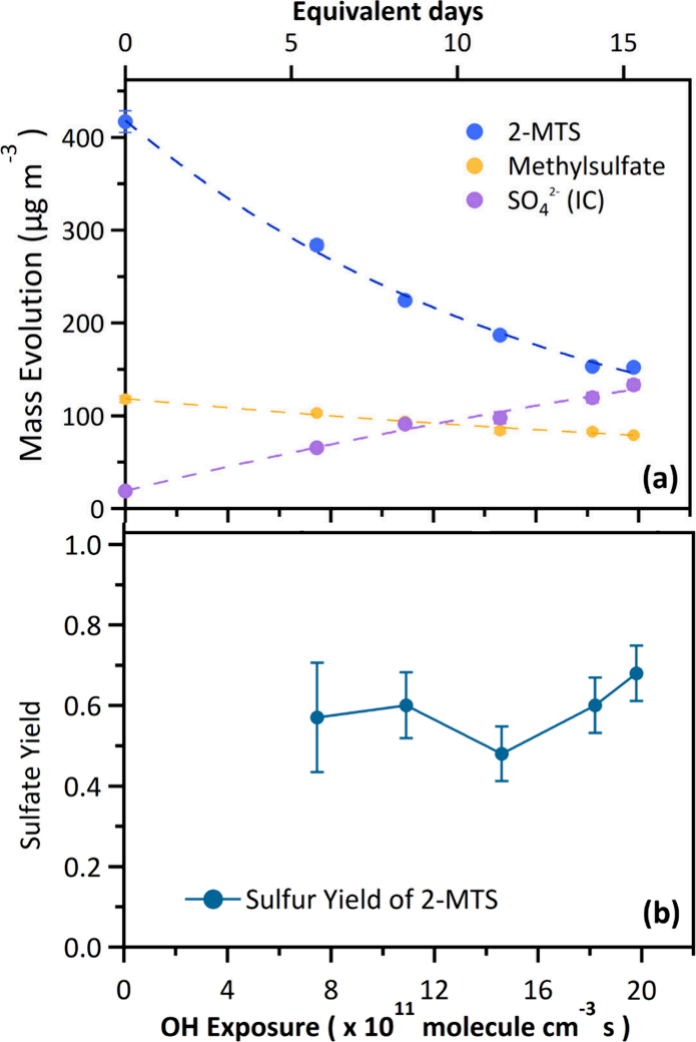
(a) Mass evolution of HILIC/ESI-HR-QTOFMS-measured 2-MTS and methylsulfate, as well as inorganic sulfate (measured by IC) upon heterogeneous ^•^OH oxidation. (b) Sulfate yields (=Δ[SO_4_^2–^]/Δ[2-MTS]) determined at different ^•^OH exposures upon heterogeneous ^•^OH oxidation of 2-MTS aerosols. The top *x*-axis shows the equivalent photochemical age in the atmosphere assuming a 24 h averaged ^•^OH concentration of 1.5 × 10^6^ molecules cm^–3^.

Consistent with our prior study,^7^ control experiments without UV or without O_3_ showed no significant loss of 2-MTS and several OS products were formed from the heterogeneous ^•^OH oxidation of 2-MTS as summarized in Table S1. Their modest signal intensities also indicate that a considerable fraction of sulfur resulting from the oxidation of 2-MTS might exist in forms other than those of organosulfates. HILIC/ESI-HR-QTOFMS extracted ion chromatograms (EICs) of 2-MTS and its major reaction products are depicted in Figures S1 and S2, respectively. In Figure S1a, before oxidation, peaks at 7.17 and 8.86 min correspond to the deprotonated molecules ([M – H]^−^) of the 2-MTS diastereomers (*m*/*z* 215). Upon heterogeneous oxidation, the intensity of 2-MTS peaks decreased (Figure S1b), and new product peaks appeared (Figure S2), representing C_5_H_7_O_7_S^–^ (*m*/*z* 211), C_5_H_9_O_7_S^–^ (*m*/*z* 213), C_5_H_9_O_8_S^–^ (*m*/*z* 229), and C_5_H_11_O_8_S^–^ (*m*/*z* 231). These results are consistent with major OS products identified in our previous work.^7^ Notably, the total intensities of these OSs were also found to be <20% of that of reacted 2-MTS upon oxidation (Figure S2). The decay of 2-MTS upon oxidation is given in Figure 1a, and its oxidation kinetics is discussed in Section S4.

In addition to the formation of new OSs, the heterogeneous ^•^OH oxidation of 2-MTS also leads to the generation of inorganic sulfates (measured as total SO_4_^2–^) as confirmed by IC analysis (Figure 1a). Figure S3 illustrates the IC chromatograms before and after oxidation. Before oxidation, two peaks were identified: Br^–^ (retention time of 13.2 min) and SO_4_^2–^ (retention time of 18.1 min). A small SO_4_^2–^ peak was present in the IC chromatogram prior to oxidation, likely originating from the ammonium sulfate in the 2-MTS standard (∼4.5 wt %) as the hydrolysis of 2-MTS was expected to be insignificant.^15,17^ Methylsulfate (CH_3_SO_4_^–^) was also present in the standard (∼25.9 wt %). We acknowledged its susceptibility to heterogeneous ^•^OH oxidation, resulting in the formation of SO_4_^2–^.^11^ Therefore, the decay of methylsulfate upon oxidation was monitored by HILIC/ESI-HR-QTOFMS (Figure 1a). Figure S4 shows the aerosol sulfur mass distribution and evolution of the sulfur-containing species mentioned above (i.e., 2-MTS, methylsulfate, OS products, and inorganic sulfates), and a sulfur balance ranging from 85% to 95% was observed over the experimental ^•^OH exposures. The uncharacterized fraction of aerosol sulfur was likely attributed to other inorganic sulfur species, such as S_2_O_8_^2–^, which may arise through the self-reaction of sulfate radicals.^11,28^ Assuming a conservative sulfate yield of one, which represents an upper bound based on a previous study,^11^ the amount of SO_4_^2–^ formed from the oxidized methylsulfate contributed approximately 22–29% of the total SO_4_^2–^ detected by IC after heterogeneous oxidation (Figure S5). Despite potential uncertainties, a notable quantity of SO_4_^2–^ was produced through heterogeneous ^•^OH oxidation of 2-MTS (Section S5), factoring in corrections for SO_4_^2–^ derived from ammonium sulfate and the heterogeneous ^•^OH oxidation of methylsulfate, as summarized in Table S2. As a result, the corrected inorganic sulfate formation was used in the following sulfate yield calculations.

### 3.2 Sulfate Yield

We observed high sulfate yields (Figure 1b), ranging from 0.48 ± 0.07 to 0.68 ± 0.07 across varying ^•^OH exposures. The sulfate yield, defined as the total number of moles of HSO_4_^–^ and SO_4_^2–^ (as corrected total sulfate) formed per mole of 2-MTS reacted, was determined for a specific ^•^OH exposure by the equation sulfate yield = Δ[SO_4_^2–^]/Δ[2-MTS]. The observation of high sulfate yields underscores that the conversion of sulfur from the oxidation of particulate 2-MTS into inorganic sulfates, HSO_4_^–^ and SO_4_^2–^, was substantial.

### 3.3 Inorganic Sulfate Formation Pathways: Sulfate/Sulfite Radical Chemistry

The reaction mechanisms that result in the generation of newly formed OSs (Table S1) during the heterogeneous ^•^OH oxidation of 2-MTS aerosols were discussed in our prior study.^7^ In the following discussion, our emphasis is on elucidating reaction pathways that lead to the formation of inorganic sulfates.

**Scheme 1:**
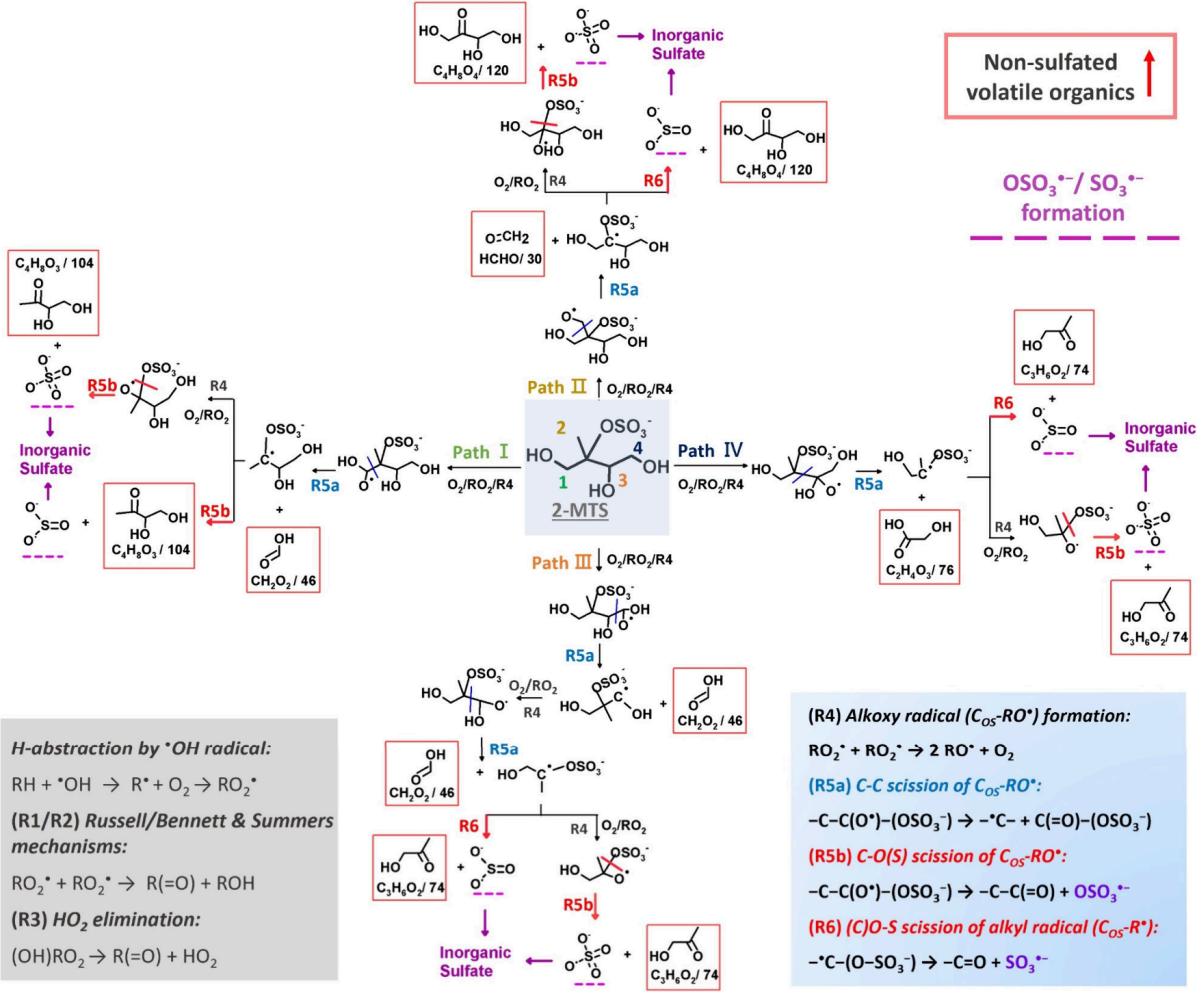
Reaction Mechanisms Tentatively Proposed for Inorganic Sulfate Formation upon Heterogeneous ^•^OH Oxidation of 2-MTS The pathways, labeled as Paths I–IV, correspond to reactions initiated from carbon sites 1–4, respectively. A detailed description for R1–R3 can be found in Section S2.

We postulate that the high sulfate yields observed can be attributed to the likely favorable formation of SO_4_^•–^ during heterogeneous ^•^OH oxidation, as illustrated in Scheme 1. The 2-MTS is highly oxygenated bearing various functional groups. Upon oxidation, the likelihood of generating an alkoxy radical (RO^•^) is high, as the spatial arrangement of two peroxy radicals (RO_2_^•^), crucial for the formation of functionalization products, is hindered by the presence of neighboring hydroxyl (-OH), methyl (-CH_3_), and sulfate ester (-OSO_3_^–^) groups.^18−21^ The high sulfate yields find partial support in the relatively low intensities of functionalization products, such as more oxygenated C_5_ OSs, as shown in Table S1. We also propose that the -OH group at the β-position facilitates the decomposition of RO^•^ by significantly decreasing the activation energy.^22−24^ Additionally, when an alkoxyl group forms at the α-position of -OSO_3_^–^, the resulting C_OS_-RO^•^ can decompose via cleavage of either a C–C bond (R5a) or a C–O(S) bond (R5b). The latter pathway (R5b) produces SO_4_^•–^, leading to inorganic sulfate formation through a series of aerosol-phase reactions (Section S2).^11^ The dissociation of a C–O(S) bond is presumed to be more energetically favorable than that of a C–C bond due to the lower dissociation energy associated with C–O(S) bonds.^25,26^ Furthermore, the fragmentation of anionic alkoxy radicals can exhibit a propensity for generating radical anion fragments as the deprotonated groups on the leaving radicals (i.e., SO_4_^•–^) can restore thermodynamic stability and lower the energy barrier for alkoxy decomposition.^27^ The negligible amounts of smaller OSs (i.e., C_2_, C_3_, and C_4_) detected by HILIC/ESI-HR-QTOFMS analysis (Table S1) may indicate the significance of R5b over R5a, resulting in the formation of a SO_4_^•–^ and a corresponding nonsulfated product.

In addition to the SO_4_^•–^ chemistry, we also tentatively propose an alternative mechanism for the formation of inorganic sulfates through sulfite radical (SO_3_^•–^) chemistry. Recent experimental and theoretical studies of organonitrate oxidations against ^•^OH have suggested that the nitrate group (-ONO_2_^–^) positioned at the β-position of an alkoxy radical can significantly promote C–C bond decomposition, and the resultant ^•^R-ONO_2_ at a transient state can spontaneously undergo O–N bond scission, releasing NO_2_, with barriers and rate constants comparable to those of direct O_2_ addition.^23,28−30^ In analogy to ^•^R-ONO_2_ chemistry, here we postulate that a β-OSO_3_^–^ alkoxy radical [-C(O^•^)–C(OSO_3_^–^)-] might decompose from the C–C bond into a α-OSO_3_^–^ alkyl radical (C_OS_-R^•^), which can sequentially fragment from the O–S bond into a nonsulfated product and a SO_3_^•–^ (R6, Scheme 1), leading to inorganic sulfate formation through a series of aerosol-phase reactions as illustrated in a previous study.^31^ Future investigations, encompassing quantum calculations and experimental studies, particularly those enabling *in situ* detection of SO_4_^•–^ and SO_3_^•–^, are warranted to assess the significance of these reaction pathways that led to the formation of inorganic sulfates upon heterogeneous ^•^OH oxidation of 2-MTS and possibly other types of OSs.

### 3.4 Formation of Volatile Nonsulfated Products: Reduction in Aerosol Mass and Size

As depicted in Scheme 1, we expect a range of volatile nonsulfated products (Table S3) to be generated alongside the formation of inorganic sulfates. Consistent with this expectation, SEMS revealed an ∼25% decrease in aerosol mass loading at the maximum ^•^OH exposure upon heterogeneous ^•^OH oxidation of 2-MTS aerosols (Figure S6). Additionally, a reduction in the surface-weighted diameter of 2-MTS aerosols was observed, decreasing from 109 ± 2 to 90 ± 1 nm (Figure S6). Due to the low volatilities of both 2-MTS and OSs, we predominantly attribute the observed decrease in aerosol mass to the generation and subsequent evaporation of volatile nonsulfated products, as evidenced by the strong correlation between aerosol mass loss and sulfate formation (Figure S7). Furthermore, IC analysis from our prior study revealed increased signal intensities of glycolate [(OH)CHCOO^–^] and formate (HCOO^–^) ions after the oxidation of 2-MTS,^7^ indicating the formation of formic acid (HCOOH) and glycolic acid (C_2_H_2_O_3_) during the oxidation process as the first- or high-generation products (Section S7). The absence of nonsulfated products in our HILIC/ESI-HR-QTOFMS analysis might be attributed to their high volatilities, dilution during sample preparation for HILIC/ESI-HR-QTOFMS analysis, and further heterogeneous degradation. Additionally, a recent investigation of the aqueous-phase oxidation of 2-MTS by ^•^OH revealed the predominant formation of volatile species such as formic acid, acetic acid, and glycolic acid, particularly at higher extents of oxidation.^17^ Thus, future research employing analytical techniques like an online chemical ionization mass spectrometer (CIMS)^32,33^ for the detection of these volatile nonsulfated products is strongly desired.

### 3.5 Atmospheric Implications

This work demonstrates a significant conversion of aerosol sulfur from its organic to inorganic form upon heterogeneous ^•^OH oxidation of 2-MTS aerosols, a prevalent component of isoprene-derived SOAs. Additionally, we demonstrate that there is significant aerosol mass loss (25%) due to the formation of volatile nonsulfated products upon oxidation. Although differences in our laboratory and ambient conditions (i.e., oxidant level, RH, and aerosol composition and properties) may introduce discrepancies in the observed heterogeneous reactivity and chemistry of 2-MTS in atmospheric aerosols (Section S8), this work provides experimental evidence that sulfur cycling between inorganic and organic forms can be induced during the multiphase chemistry of isoprene and ^•^OH, which is particularly important in governing aerosol physicochemical properties (i.e., acidity, water uptake, and cloud condensation activity) as sulfur in its inorganic and organic forms exhibits distinct properties.^34−37^ For instance, compared to inorganic sulfates, 2-MTS exhibits a lower hygroscopicity^36^ and a slightly weaker acidity in neutral forms.^38^ Thus, the aerosol water uptake behavior and acidity is expected to change during the multiphase formation and oxidation of 2-MTS aerosol, which can further affect the gas-particle partitioning of volatile organic species^39^ and ultimately the aerosol mass loading. Moreover, the findings of this study have implications that can be extended to the heterogeneous oxidation of other structurally similar OSs found in isoprene-derived SOA. It is expected to modify the physicochemical properties of isoprene-derived SOA, potentially impacting their environmental and climatic consequences, thereby warranting further investigation.

## Data Availability

Data are available upon request from the corresponding authors.

## Supporting Information Available

Additional experimental details of the identification and quantification of organosulfates (Section S1), description of generic reaction mechanisms (Section S2), analytical details of sulfate formation (Section S3), oxidation kinetics (Section S4), details of the correction for sulfate formation from 2-MTS oxidation (Section S5), calculation of uncertainties (Section S6), discussion of the formation of formic acid and glycolic acid (Section S7), and discussion of the heterogeneous reactivity and chemistry of 2-MTS in atmospheric aerosols (Section S8) (PDF)
