# Supplementary material for: Formation of Inorganic Sulfate and Volatile Nonsulfated Products from Heterogeneous Hydroxyl Radical Oxidation of 2-Methyltetrol Sulfate Aerosols: Mechanisms and Atmospheric Implications
Source: Environ Sci Technol Lett. 2024 Aug 7;11(9):968–74. doi: 10.1021/acs.estlett.4c00451 (PMC11391575; doi:10.1021/acs.estlett.4c00451)
Supplement: Supplementary file 1 — ez4c00451_si_001.pdf [file ez4c00451_si_001.pdf]

# Supporting Information

# Formation of Inorganic Sulfate and Volatile Non-Sulfated Products from Heterogeneous Hydroxyl Radical Oxidation of 2-Methyltetrol Sulfates Aerosols: Mechanisms and Atmospheric Implications

Rongshuang Xu[1], Yuzhi Chen[2,3], Sze In Madeleine Ng[4], Zhenfa Zhang[2], Avram Gold[2], Barbara J. Turpin[2], Andrew P. Ault[5*], Jason D. Surratt[2,6*] and Man Nin Chan,[4,7*]

[1]School of Ecology and Applied Meteorology, Nanjing University of Information Science & Technology, Nanjing 210044, China

[2]Department of Environmental Sciences and Engineering, Gillings School of Global Public Health, University of North Carolina at Chapel Hill, Chapel Hill, North Carolina, United States 27599

[3]Atmospheric, Climate, and Earth Sciences, Pacific Northwest National Laboratory, Richland, Washington 99352, United States

[4]Earth System Science Programme, Faculty of Science, The Chinese University of Hong Kong, Hong Kong, China Department of Chemistry, The Hong Kong University of Science and Technology, Hong Kong, China

[5]Department of Chemistry, College of Literature Sciences and the Arts, University of Michigan, Ann Arbor, Michigan, United States 48109

[6]Department of Chemistry, College of Arts and Sciences, University of North Carolina at Chapel Hill, Chapel Hill, North Carolina, United States 27599

[7]The Institute of Environment, Energy, and Sustainability, The Chinese University of Hong Kong, Hong Kong, China

**Corresponding authors:*

Man Nin Chan, mnchan@cuhk.edu.hk

Jason D. Surratt, surratt@unc.edu

Andrew P. Ault, aulta@umich.edu

## Section S1: HILIC/ESI-HR-QTOFMS analysis for characterization of OSs.

A 50 µL aliquot of each PILS sample was drawn and diluted in 950 µL acetonitrile (ACN, HPLC grade, Fisher Scientific) after collection in order to achieve the solvent composition of the organic mobile phase. The aliquots were then immediately stored in the dark at −20 °C prior to HILIC/ESI−HR-QTOFMS analysis. 2-MTS and OSs were characterized using an Agilent 6500 Series UPLC system coupled with an electrospray ionization (ESI) source and a Quadrupole-Time-of-Flight Mass Spectrometer (Agilent 6250). The aliquot was injected into a Waters ACQUITY UPLC ethylene bridged hybrid amide (BEH-Amide) column (2.1 × 100 mm, 1.7-µm particle size, Waters) at 35 °C. The mobile phases consisted of two sets of eluents: eluent (A) containing 0.1% (w/w) of ammonium acetate in $H_2O$ solution, and eluent (B) containing 0.1% (w/w) of ammonium acetate in an ACN-$H_2O$ solution (95:5 vol/vol). Both eluents were adjusted to a pH level at 9.0 using $NH_4OH$ (TraceMetal Grade, Fisher Scientific). The eluent gradient was 0% of eluent A for initial 4 min, then increased to 5.6% A over next 6 min and held constant for another minute, decreased to 0% from 11 min to 11.5 min, and held constant till 15 min. The ESI source within the mass spectrometer was operated in negative ion mode, and the mass spectra were recorded from mass-to-charge ratio (*m/z*) 60 to 1000 at high-resolution mode (4GHz). Data was analyzed by Mass Hunter Version B.06.00 Build 6.0.633.0 software (Agilent Technologies).

Detected OSs are summarized in **Table S1**. As a first approximation, an estimation for the abundance of these newly formed OS products relative to reacted 2-MTSs (in the term of OS yield) were also derived based on their signal intensities and corresponding responses factors (RFs) in HILIC/ESI−HR-QTOFMS analysis:

$$yield_{i,j} = {\Delta_{intensity[OS_i]_j} \times RF_i} \Big/ {\Delta_{intensity[2-MTS]_j} \times RF_{2-MTS}} \quad \textbf{(Eqn. S1)}$$

where $\Delta_{intensity[OS_i]_j}$ refers to detected change in the signal intensity (peak area) of a certain OS species ($OS_i$) at a given OH exposure, *j*; $RF_i$ is the RF of $OS_i$ during HILIC/ESI−HR-QTOFMS analysis. Considering the correlation between RFs of various OS standards and their retention times (RTs) elucidated in previous study,[1] the RF of specific OS species in this study was approximated to that of either methylsulfate or 2-MTS (**Table S1**), depending on which compound exhibited the closest RT. The quantification of 2-MTS and methylsulfate (a tracer in the standard) was based on the calibration curve of 2-MTS and methylsulfate standards in HILIC/ESI−HR-QTOFMS (methylsulfate sodium salt, Sigma Aldrich, Q100).

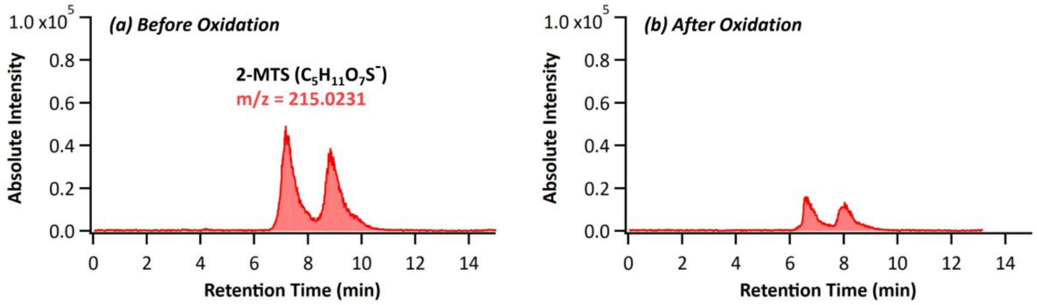


**Figure S1.** The extracted ion chromatograms (EICs) of 2-MTS detected by HILIC/ESI-HR-QTOFMS before heterogeneous ${}^{\bullet}$OH oxidation (a) and at the maximum ${}^{\bullet}$OH exposure (b).

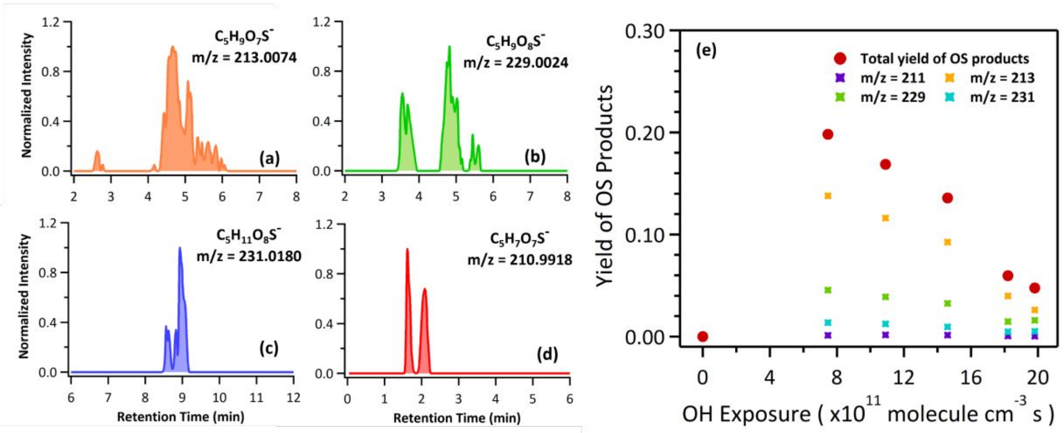


**Figure S2.** The extracted ion chromatograms (EICs) detected by HILIC/ESI-HR-QTOFMS in negative ion mode, and the formation evolution profile of major $C_5$ OSs formed upon heterogeneous ${}^{\bullet}$OH oxidation of 2-MTS (only major OS products with yield larger than 0.001 are presented in this figure, other OS products can be found in **Table S1**).

**Section S2: The generic reaction mechanism of heterogeneous $^{\bullet}$OH oxidation of organosulfates (OSs) involving the inorganic sulfates formation**

Generally, the chemical transformation of organics can proceed both by the addition of a new functional groups into parent molecules (functionalization) and the fragmentation of the carbon skeleton via chemical bond scission (fragmentation). As shown in **Scheme S1,** heterogeneous $^{\bullet}$OH oxidation of OSs initiates with hydrogen atom abstraction from a C-H bond, leading to the formation of an alkyl radical ($R^{\bullet}$), which can instantly react with an oxygen ($O_2$) molecule to form a peroxyl radical ($RO_2^{\bullet}$). The self- or cross-reactions of $RO_2^{\bullet}$ can result in two carbonyl products (**R1**),[2] an alcohol product and a carbonyl product (**R2**),[3] and α-hydroxylperoxy radical can undergo unimolecular $HO_2^{\bullet}$ elimination (**R3**).[4] Simultaneously, self- or cross-reactions of $RO_2^{\bullet}$ can form alkoxy radicals ($RO^{\bullet}$) (**R4**), which can undergoes C-C bond cleavage, forming smaller fragmentation products (**R5a**).[5-7] Meanwhile, when the $–OSO_3^-$ group is present at the α-position of the $RO^{\bullet}$, this α-$OSO_3^-$ alkoxy radical (hereby denoted as $C_{OS}$-$RO^{\bullet}$) can decompose through the cleavage of the C-O(S) bond (**R5b**) to yield a non-sulfated product and a $SO_4^{\bullet-}$, which can subsequently initiate a series of aerosol-phase reactions to produce to inorganic sulfates (**RS1–RS3**).[8-10]

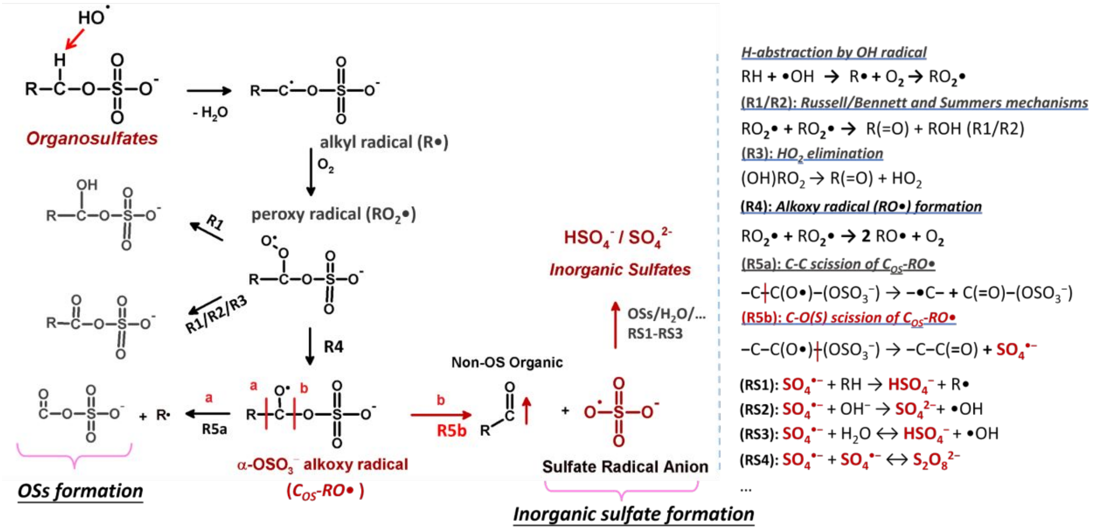


**Scheme S1**. A simplified reaction scheme proposed for the heterogeneous $^{\bullet}$OH oxidation of OSs.

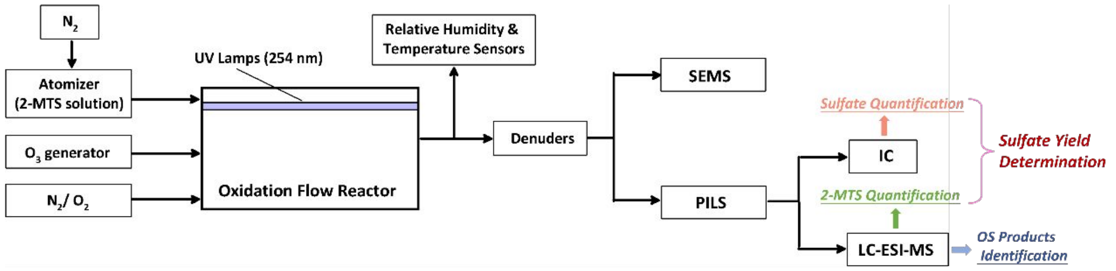


**Scheme S2.** A schematic diagram of experimental setup and chemical analysis for the heterogeneous $^{\bullet}$OH oxidation of 2-MTS aerosols.

## Section S3: IC analysis for quantification of inorganic sulfates

The amount of inorganic sulfate ($SO_4^{2-}$) formed upon heterogeneous oxidation was quantified using the IC method. A 25 μL of each PILS sample was injected into an IC system (ICS 3000, Thermo Fisher) equipped with an AS11-HC guard column (IonPac, 2×50 mm, Thermo Scientific) and anion-exchange column (2×250 mm, Thermo Scientific). The temperature of the conductivity detector (Dionex) interfaced to the IC was set at 35 °C. The 35-min potassium hydroxide (KOH) eluent program utilized for the IC was as follows: KOH was increased from 1 mM to 30 mM KOH from 0 to 25 min, then ramped to 84 mM KOH from 25 to 30 min, decreased to 1 mM until 30.1 min and held constant until 35 min. The quantification of eluent peaks was based on calibration chromatograms of ammonium sulfate standards (Sigma Aldrich, purity ⩾ 99.0%). It is known that bisulfate ion ($HSO_4^-$) is being converted into $SO_4^{2-}$ upon mixing with the alkaline eluent.[10, 11] As a result, the concentration of $SO_4^{2-}$ quantified by the IC method represents a total amount of $HSO_4^-$ and $SO_4^{2-}$ produced upon heterogeneous oxidation at a given $^{\bullet}OH$ exposure. In addition, peroxydisulfate ion ($S_2O_8^{2-}$) could be a potential product resulting from sulfate/sulfite radical reaction (**RS4, Scheme S1**). Our recent study has confirmed the $S_2O_8^{2-}$ formation in IC chromatograms as a result of the heterogenous oxidation of another organosulfur species (hydroxymethanesulfonate, $(OH)CH_2SO_3^-$) using 200 mM of NaOH as the eluent in IC.[11] We acknowledge that the detection of peroxydisulfate ($S_2O_8^{2-}$) could provide a more comprehensive information of the distribution and evolution of aerosol sulfur mass during the oxidation of 2-MTS aerosols (**Figure S4**). However, the IC analytical method in this study was primarily employed for the quantification of inorganic sulfate and did not have the capability to detect the presence of $S_2O_8^{2-}$.

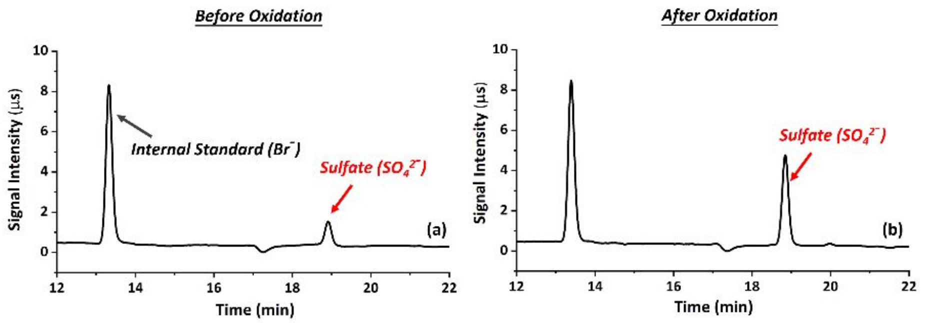


**Figure S3.** Ion chromatograms of 2-MTS aerosols before (a) and after (b) heterogeneous oxidation.

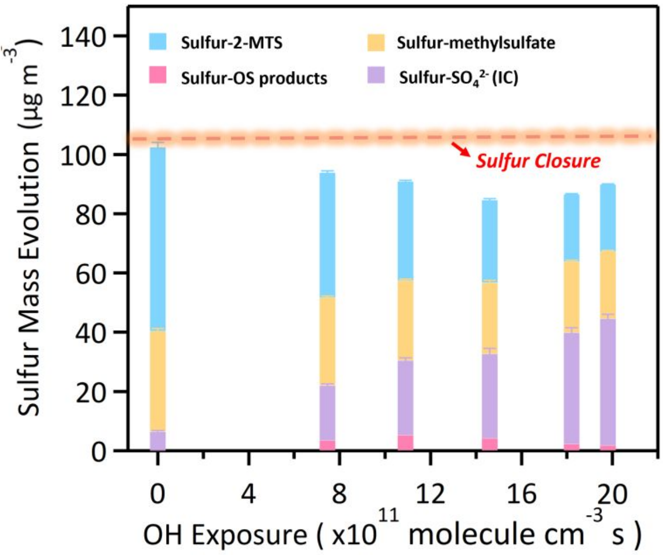


**Figure S4.** The sulfur mass distribution and evolution of sulfur-containing species including 2-MTS, methylsulfate, OSs products and inorganic sulfates upon the oxidation of 2-MTS aerosols (The blue bars represent the sulfur mass attributed to 2-MTS molecules, and the sulfur mass associated with OS products is determined based on the estimated abundance of OSs, calculated using the methodology outlined in **Section S1**).

## Section S4: Oxidation kinetics of 2-MTS

Based on the aerosol composition data characterized at different extents of oxidation, 2-MTS was found to be oxidized by $^{\bullet}$OH at a significant rate **(Figure 1a)**. Approximately 64% of 2-MTS was reacted at the highest $^{\bullet}$OH exposure. Heterogeneous oxidation kinetics can be quantified by measuring the decay of 2-MTS over the $^{\bullet}$OH exposure, and expressed by the effective second-order heterogeneous $^{\bullet}$OH rate constant, *k*:

$$ln\frac{I}{I_0} = -k \times [OH] \times t \quad \textbf{(Eqn. S2)}$$

where $I$ is the signal intensity of 2-MTS at a given $^{\bullet}$OH exposure, $I_0$ is its signal intensity before oxidation, and $[OH] \times t$ is the $^{\bullet}$OH exposure. The $k$ was fitted to be $5.36 \pm 0.11 \times 10^{-13}$ $cm^3$ $molecule^{-1}$ $s^{-1}$ for 2-MTS aerosols with a surface-weighted diameter of 109 ± 2 nm, and was in a good agreement with our previous work ($k = 4.9 \pm 0.6 \times 10^{-13}$ $cm^3$ $molecule^{-1}$ $s^{-1}$ for an aerosol size of 119 ± 4 nm).[12] Based on the kinetic data, the e-folding lifetime of 2-MTS against heterogeneous $^{\bullet}$OH oxidation, $\tau = 1/k[OH]$, is estimated to be 14.4 ± 0.3 days assuming the ambient $^{\bullet}$OH concentration of $1.5 \times 10^6$ molecules $cm^{-3}$. Given atmospheric aerosols of a comparable size that exhibit an average lifetime of 5–12 days against wet or dry deposition,[13] heterogeneous $^{\bullet}$OH oxidation of 2-MTS could be a comparable removal processes and warrants attention during the simulation of chemical transport models to better predict the atmospheric abundance of 2-MTSs.

## Section S5: Correction for inorganic sulfate formation from the 2-MTS oxidation

### 5.1 Impurities in synthesised 2-MTS standard

The 2-MTS standard ($C_5H_{12}O_7S^-$, counter ion $NH_4^+$) investigated in this work was synthesized following previously published procedure[14] and its purity was determined to be 60.2% using proton nuclear magnetic resonance ($^1H$ NMR) spectroscopy with impurities including 25.9% (w/w) of methylsulfate ($CH_3SO_4^-$), 4.5% of ammonium sulfate ($(NH_4)_2SO_4$, AS) and $C_2$-$C_4$ OSs accounting for the residual mass.[12] Here, the sulfate detected by IC before oxidation ([$SO_4^{2-}$-AS]) was attributed to the presence of $(NH_4)_2SO_4$ as shown in **Figure S3a**. It is also acknowledged that the heterogenous $^{\bullet}OH$ oxidation of methylsulfate can also lead to the inorganic sulfate formation.[8, 10] However, its existence in 2-MTS standard cannot fully account for the significant increase in inorganic sulfate signal observed by IC. Assuming a conservative sulfate yield of one, the amount of $SO_4^{2-}$ formed from the oxidized methylsulfate contributed approximately 22.2% - 28.7% of the total $SO_4^{2-}$ detected by IC after oxidation (**Figure S5**). Despite the potential uncertainties, these results suggest the presence and further reactions of the trace impurities could not fully explain the observed inorganic sulfate formation (**Table S2**) and was corrected for the quantification of sulfates from 2-MTS oxidation using **Eqn. S3**:

$$\left[SO_4^{2-}\text{-corrected}\right]_i = \left[SO_4^{2-}\text{-IC}\right]_i - \left[SO_4^{2-}\text{-}CH_3SO_4^-\right]_i - \left[SO_4^{2-}\text{-AS}\right] \quad \textbf{(Eqn. S3)}$$

where the $\left[SO_4^{2-}\text{-corrected}\right]_i$, $\left[SO_4^{2-}\text{-IC}\right]_i$ and $\left[SO_4^{2-}\text{-}CH_3SO_4^-\right]_i$ are the corrected inorganic sulfate formation resulting from 2-MTS oxidation, the total inorganic sulfate amount measured by IC and the inorganic sulfate formed from methylsulfate oxidation derived from its decay at a given $^{\bullet}OH$ exposure, respectively. This corrected inorganic sulfate formation was used for the determination of sulfate yields.

### 5.2 Hydrolysis of OS species

It is noted that the acid-catalysed hydrolysis of OSs in aqueous phase can lead to the formation of polyols and $H_2SO_4$.[15, 16] Recent research has indicated that the lifetime of 2-MTSs against hydrolysis is approximately 28 days in a neutral solution and 10 days in a 0.1 M sulfuric acid solution with a pH below 1.[17] In our experimental setup, the aerosol residence time in the OFR was maintained at approximately 128 s for all $^{\bullet}OH$ exposure levels. After oxidation, the aerosol particles were collected onto PILS and subsequently analyzed within two weeks or less using IC and HILIC/ESI-HR-QTFOMS. Given that, it is expected that the hydrolysis of 2-MTS and newly formed OSs product would likely have a negligible impact on sulfate formation during such shorter timescales. In addition, the hydrolysis of OS species can lead to the formation of polyols. For instance, xylitol ($C_5H_{12}O_5$) can be formed through the hydrolysis of major OSs products (i.e., $C_5H_{11}O_8S^-$). This xylitol has a low volatility with saturation vapor pressure of $1.71 \times 10^{-6}$ Pa,[18] comparable to that of organosufates[19] and thus tends

to stay in aerosol phase after formation. Consequently, the absence of these $C_5$ polyol species and other alcohol products in our mass spectra could support the proposition that the hydrolysis of these OSs holds minor significance in the sulfate formation. More importantly, an obvious increase in the amount of inorganic sulfates with increasing $^{\bullet}$OH exposure levels, as demonstrated in **Figure S4** and **S5**, is observed and likely a result of heterogenous reactions. Taken together, although we could not completely rule out the possibility of the formation of sulfate via the hydrolysis of OS products, the enhanced inorganic sulfates formation is anticipated to be mainly due to the heterogenous $^{\bullet}$OH reactions of 2-MTS.

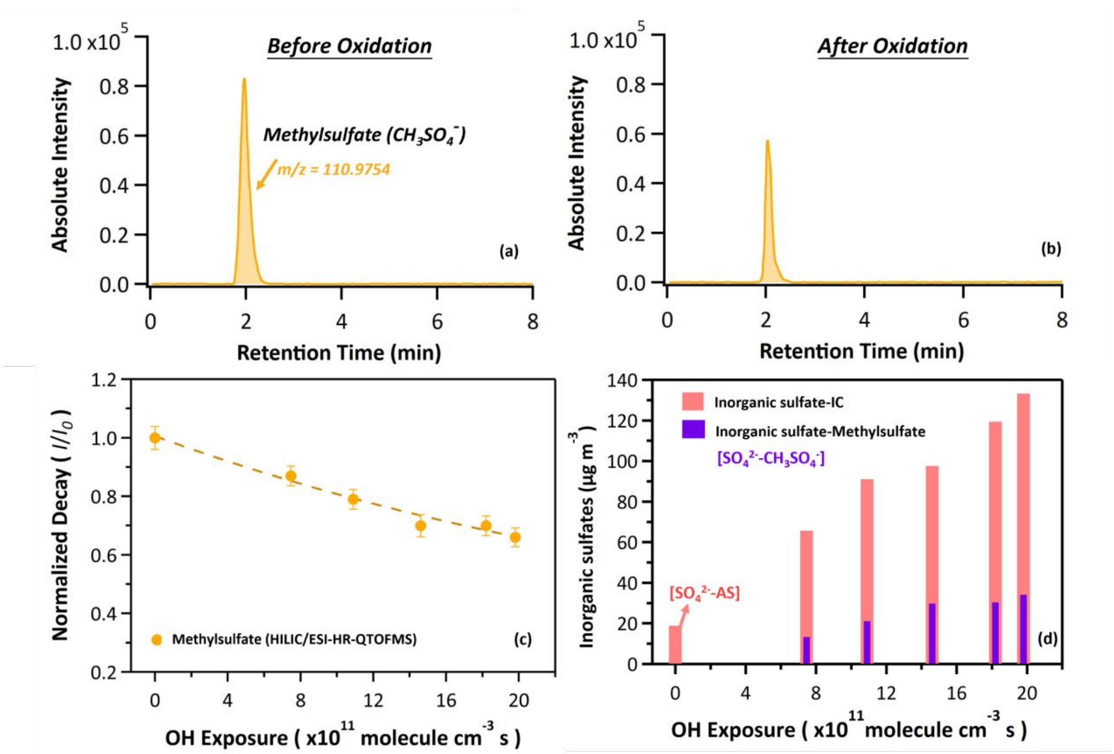


**Figure S5.** The extracted ion chromatograms (EICs) of methylsulfate from HILIC/ESI-HR-QTOFMS analysis in negative ion mode before (a) and after (b) heterogeneous $^{\bullet}$OH oxidation (RT = 2.03 min); (c) the normalized decay of methylsulfate upon heterogenous $^{\bullet}$OH oxidation; (d) the total amount of $SO_4^{2-}$ at different $^{\bullet}$OH exposures measured by IC (pink bar) and the amount of $SO_4^{2-}$ formed from heterogeneous $^{\bullet}$OH oxidation of methylsulfate (purple bar).

## Section S6: Uncertainty determination

### 6.1 Uncertainty for the quantification of 2-MTS, methylsulfate and $SO_4^{2-}$

Measurement precisions for the concentration of species $i$ ($\sigma_{Ci}$) are propagated from precisions of volumetric measurements, chemical composition measurements, and blank sample variability and sample repeatability.[20] For simplicity, the following equations are used to calculate the uncertainty associated with our PILS sample solutions:

$$C_i = \frac{M_i - B_i}{V} \quad (1)$$

$$B_i = \frac{1}{n}\sum_{j=1}^{n} B_{ij} \;\; for\; B_i > \sigma_{B_i} \quad (2)$$

$$B_i = 0 \;for\; B_i \leq \sigma_{B_i} \quad (3)$$

$$\sigma_{B_i} = STD_{B_i} = \left[\frac{1}{n-1}\sum_{j=1}^{n}(B_{ij} - B_i)^2\right]^{\frac{1}{2}} \;for\; STD_{B_i} > SIG_{B_i} \quad (4)$$

$$\sigma_{B_i} = STD_{B_i} = \left[\frac{1}{n}\sum_{j=1}^{n}(\sigma_{B_{ij}})^2\right]^{\frac{1}{2}} \;for\; STD_{B_i} \leq SIG_{B_i} \quad (5)$$

$$\frac{\sigma_V}{V} = 0.05 \quad (6)$$

$$\sigma_{C_i} = \left[\frac{{\sigma_{M_i}}^2 + {\sigma_{B_i}}^2}{V^2} + \frac{{\sigma_V}^2 (M_i - B_i)^2}{V^4}\right]^{\frac{1}{2}} \quad (7)$$

where

$B_i$ = average amount of species $i$ in blank samples

$B_{ij}$ = the amount of species $i$ found in blank sample $j$

$C_i$ = the concentration of species $i$

$M_i$ = amount of species $i$ in sample solution

$n$ = total number of samples in the sum

$SIG_{Bi}$ = the root mean square error (RMSE), the square root of the averaged sum of the squared $\sigma_{Bij}$

$STD_{Bi}$ = standard deviation of the blank samples

$\sigma_{Bi}$ = blank precision for species $i$

$\sigma_{Bij}$ = precision of the species $i$ found in blank sample $j$

$\sigma_{Ci}$ = propagated precision for the concentration of species $i$

$\sigma_{Mi}$ = precision of amount of species $i$

$\sigma_V$ = precision of sample volume

$V$ = sample volume (25 μL for IC analysis, 50 μL for LC/ESI-HR-MS/MS analysis)

The precisions ($\sigma_{Mi}$) were determined from duplicate analysis of samples. When duplicate sample analysis is made, the range of results, $R$, is nearly as efficient as the standard deviation since two measures differ by a constant ($1.128\sigma_{Mi} = R$). Based on the blank samples and duplicate samples, coefficients needed for determining uncertainty are given in following table:

| Species | Quantification method | No. of Blanks | No. of duplicate standard | Blank Precision ($\sigma_{Bi}$, μg) | Duplicate Precision ($\sigma_{Mi}$, μg) |
|---|---|---|---|---|---|
| 2-MTS | HILIC/ESI-HR-QTOFMS | 2 | 2 | 0.0027 | 0.0223 |
| Methylsulfate | HILIC/ESI-HR-QTOFMS | 2 | 2 | 0.0013 | 0.0136 |
| Sulfate/bisulfate | IC | 2 | 2 | 0.0003 | 0.0001 |

### 6.2 Uncertainty for the sulfate yield

Based on the calculation of sulfate yield (**Eqn. S4**) and the uncertainties propagated from measurement precisions, the uncertainty for sulfate yield can be calculated from **Eqn. S5**.

$$sulafte\ yield = \frac{\Delta[\mathrm{SO_4^{2-}\text{-}corrected}]_i}{\Delta[2\mathrm{-MTS}]_i} = \frac{(\Delta[\mathrm{SO_4^{2-}\text{-}IC}]_i - \Delta[\mathrm{SO_4^{2-}\text{-}CH_3SO_4^-}]_i)}{\Delta[2\mathrm{-MTS}]_i} \quad \textbf{(Eqn. S4)}$$

$$\sigma_{yield_j} = \left[\frac{(\sigma_{2-\mathrm{MTS}_j}{}^2\left([\mathrm{SO_4^{2-}\text{-}IC}]_0 - [\mathrm{SO_4^{2-}\text{-}CH_3SO_4^-}]_0 - [\mathrm{SO_4^{2-}\text{-}IC}]_j + [\mathrm{SO_4^{2-}\text{-}CH_3SO_4^-}]_j\right) + \left(\sigma_{[\mathrm{SO_4^{2-}\text{-}IC}]_0}{}^2 + \sigma_{[\mathrm{SO_4^{2-}\text{-}IC}]_j}{}^2 + \sigma_{[\mathrm{SO_4^{2-}\text{-}CH_3SO_4^-}]_j}{}^2\right)}{(2\mathrm{-MTS}_0 - 2\mathrm{-MTS}_j)^4}\right]^{\frac{1}{2}}$$

**(Eqn. S5)**

where

$\sigma_{yield_j}$= precision of sulfate yield on sample $j$

$\sigma_{\mathrm{SO_4^{2-}\text{-}IC}_j}$= precision of sulfate on sample $j$ measured by IC

$\sigma_{\mathrm{SO_4^{2-}\text{-}IC}_0}$= precision of sulfate on first sample (prior to oxidation) measured by IC

$\sigma_{\mathrm{CH_3SO_4^-}_j}$= precision of $\mathrm{CH_3SO_4^-}$ on sample $j$ measured by HILIC/ESI-HR-QTOFMS

$\sigma_{\mathrm{CH_3SO_4^-}_0}$= precision of $\mathrm{CH_3SO_4^-}$ on first sample measured by HILIC/ESI-HR-QTOFMS

$\sigma_{2-MTS_j}$= precision of 2-MTS on sample $j$ measured by HILIC/ESI-HR-QTOFMS

$\sigma_{2-MTS_0}$= precision of 2-MTS on first sample measured by HILIC/ESI-HR-QTOFMS.

## Section S7: Formation of formic acid and glycolic acid

IC analysis from our prior study observed increased signal intensities of glycolate ((OH)CHCOO$^-$) and formate (HCOO$^-$) ions in the chromatograms after the oxidation of 2-MTS,[12] indicating the formation of formic acid (HCOOH, FA) and glycolic acid ($C_2H_2O_3$, GA) during the heterogeneous $^{\bullet}$OH oxidation of 2-MTS as elucidated in **Scheme 1**. In addition, given the high concentration of $^{\bullet}$OH in the reactor, non-sulfated organic products (i.e., $C_3H_6O_2$, $C_4H_8O_3$ and $C_4H_8O_4$) can undergo further heterogeneous oxidation and lead to the formation of FA and GA as higher-generation products. Among these organics, $C_4H_8O_4$ exhibits lower volatility (**Table S3**), making it more susceptible to $^{\bullet}$OH oxidation. Take the $C_4H_8O_4$ as an example, 3 carbon sites (carbon 1, 3, 4) of the $C_4H_8O_4$ are available for hydrogen abstraction by $^{\bullet}$OH to initiate the oxidation process. As shown in **Scheme S3**, both FA and GA can be formed during the further $^{\bullet}$OH oxidation of $C_4H_8O_4$. In a similar manner, the $^{\bullet}$OH oxidation of $C_3H_6O_2$, $C_4H_8O_3$ can contribute to the production of FA and GA. Therefore, the absence of these non-sulfated organic products in the HILIC/ESI-HR-QTOFMS analysis, along with the enhanced production of FA and GA detected in the IC analysis, could be partly explained by the occurrence of further $^{\bullet}$OH oxidation of these non-sulfated organic products leading to higher generation products.

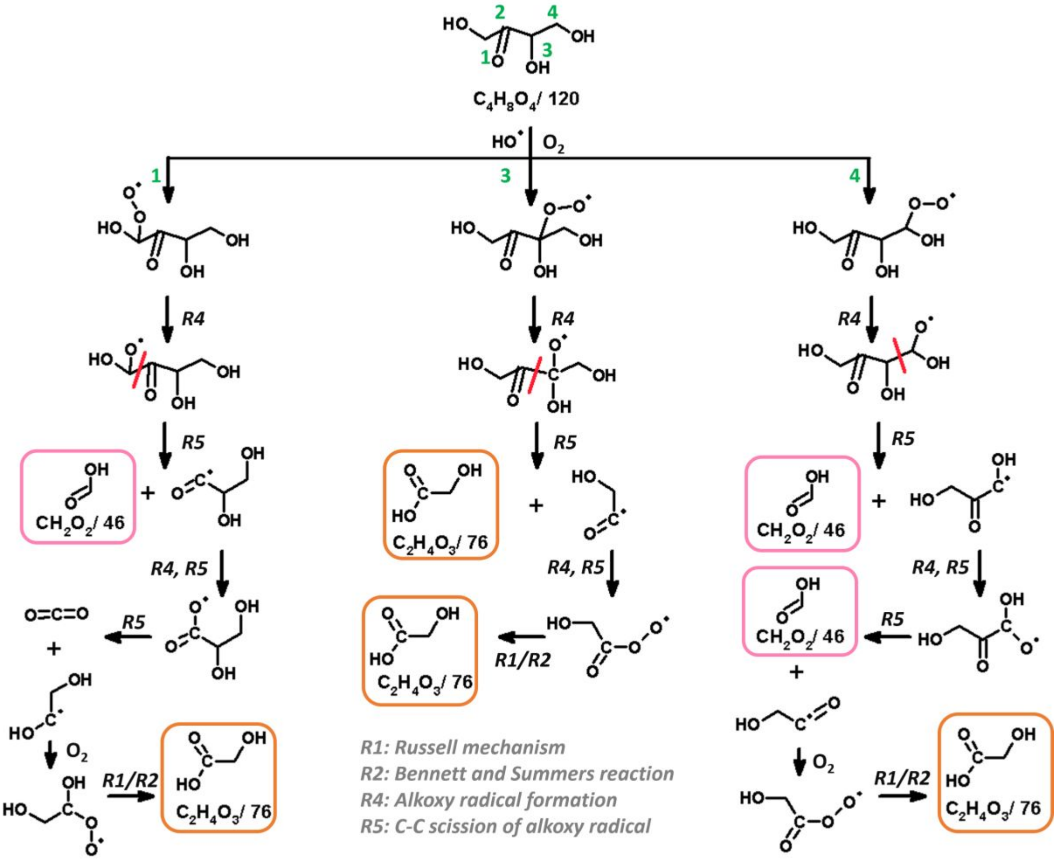


**Scheme S3**. Proposed mechanisms for formic acid ($CH_2O_2$) and glycolic acid ($C_2H_4O_3$) formation resulted from further heterogenous $^{\bullet}$OH oxidation of non-sulfated organic products (i.e., $C_4H_8O_4$).

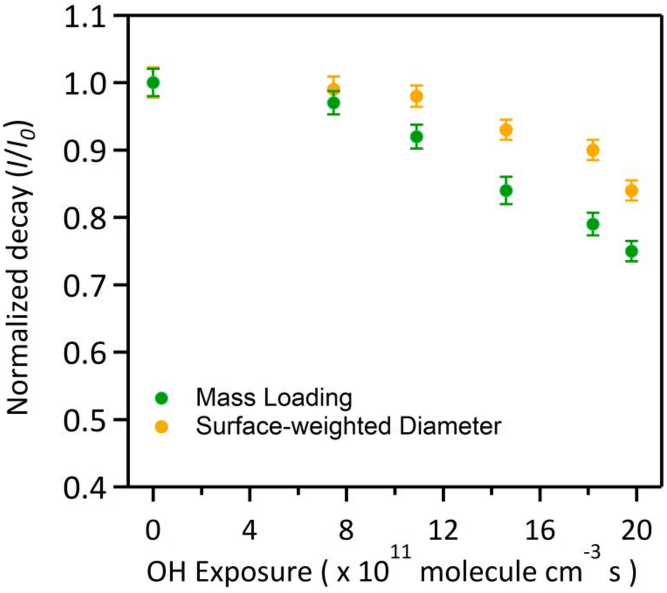


**Figure S6**. The normalized decay of aerosol mass loading and surface-weighted diameter measured by SEMS upon heterogeneous $^{\bullet}$OH oxidation of 2-MTS aerosols, starting from the initial mass loading of 393 ± 6 μg $m^{-3}$ and diameter of 109 ± 2 nm before oxidation. Here the aerosol mass loading was monitored assuming a unit density of 1 g $cm^{-3}$ for 2-MTS aerosols upon oxidation as a first approximation.

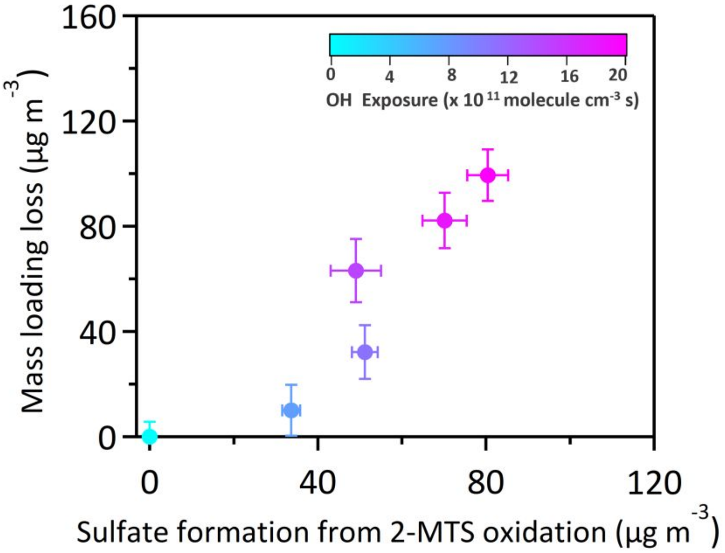


**Figure S7**. The correlation between aerosol mass loss (measured by SEMS) and sulfate formation (measured by IC) resulted from the heterogeneous $^{\bullet}$OH oxidation of 2-MTS aerosols.

**Section S8: The heterogenous reactivity and chemistry of 2-MTS in atmospheric aerosols**

This work investigated the kinetic and chemistry for heterogeneous $^{\bullet}OH$ oxidation of aqueous 2-MTS aerosol at a single RH (~60%). However, the difference in our laboratory and ambient reaction conditions may introduce discrepancies in observed reaction kinetic and sulfate yield for heterogenous $^{\bullet}OH$ oxidation of 2-MTS in atmospheric aerosols. In the following discussion, we address the possible differences and their potential impacts on the heterogeneous reaction kinetics and mechanisms of atmospheric 2-MTS aerosol.

**8.1 Low oxidant ($^{\bullet}OH$) level**

Firstly, the OFR experiments typically employ a higher concentration of oxidants to simulate long-term atmospheric oxidation processes within a shorter experimental timescale by assuming that reaction time and oxidant concentration are interchangeable.[7, 21] In this work, the $^{\bullet}OH$ concentration ($5.86 \times 10^{10}$ molecules cm$^{-3}$ to $1.54 \times 10^{10}$ molecules cm$^{-3}$) was significantly higher than ambient $^{\bullet}OH$ concentration of $1.5 \times 10^{6}$ molecules cm$^{-3}$,[22] which may introduce difference in the heterogeneous oxidation chemistry such as sulfate yield between our experiments and the real atmosphere. As shown in **Scheme 1**, the inorganic sulfate formation requires the formation of alkoxy radical ($RO^{\bullet}$) though the self- or cross-reactions of peroxy radical ($RO_2^{\bullet}$) (**R4**). A recent study has suggested that branching ratios for $RO^{\bullet}$ formation resulted from self- or cross-reactions of $RO_2^{\bullet}$ could be smaller under lower $RO_2^{\bullet}$ concentration.[23] Given that the formation rate and concentration of $RO_2^{\bullet}$ would be lower under low $^{\bullet}OH$ level conditions, it is expected that the yields of inorganic sulfate reported in this work would be higher than those observed in the atmosphere. Additionally, in the atmosphere, bimolecular reactions with $HO_2$[24] or NOx[25] may also compete with the self- or cross-reactions of $RO_2^{\bullet}$ although $RO^{\bullet}$ is more likely formed from the $RO_2^{\bullet}$ + NO reactions and subsequently undergoes bond dissociation. Therefore, future laboratory or modeling investigations are highly desired to explore the effect of $^{\bullet}OH$ concentration on the kinetic and inorganic sulfate formation of heterogeneous $^{\bullet}OH$ oxidation of 2-MTS considering the presence or absence of $HO_2^{\bullet}$ or NOx.

**8.2 Atmospheric humidity condition**

In addition, the atmospheric humidity condition (i.e., relative humidity, RH) has been suggested to impact the heterogenous reaction rates of OSs,[8, 10] primarily by influencing the aerosol liquid water content. Laboratory study has reported that aerosols containing the ammonium salt of 2-MTS can absorb or desorb water reversibly when the RH increase or decrease in the range of 10%-90% but did not show a distinct phase transition,[26] suggesting that these aerosols likely exist in an aqueous state when RH exceeds 10%. Regarding the RH effects on the heterogenous reactivity of 2-MTS, we

anticipate two main influences. On one hand, under higher RH conditions, enhanced water uptake leads to a decrease in the bulk and surface concentration of 2-MTS and dissolved $^{\bullet}$OH. Consequently, the collision probability between 2-MTS and $^{\bullet}$OH in the bulk solution and/or on the aerosol surface decreases, thereby slowing down the overall oxidation rate. On the other hand, as RH decreases to a certain extent, the aerosol particles become highly concentrated. This high concentration may lead to a reduction in the reaction rate, as the viscosity of aerosols generally increases with solute concentration. The increased viscosity hampers the diffusion of 2-MTS within the aerosol and towards the aerosol surface for oxidation, thereby lowering the overall reactivity. As a result, the formation rate of inorganic sulfates and volatile non-sulfated products is expected to decrease or increase with the heterogenous reaction rate while the sulfate yield may be not significantly affected as it describes the formation of inorganic sulfates relative to the reaction of 2-MTS. These hypotheses can find support in our previous study which examined the effect of RH (from 75 to 85%) on the oxidative kinetics and sulfate yield of methylsulfate upon heterogeneous $^{\bullet}$OH oxidation based on experimental measurement and model simulation.[8, 10] We found higher RH could lower the overall reaction rate between methylsulfate aerosol and $^{\bullet}$OH through dilution effect while the sulfate yield is not very sensitive to the RH change. Further study is desired to fully elucidate how and to what extent RH changes alter the heterogeneous reactivity of 2-MTS and sulfate yield.

### 8.3 Aerosol composition and physicochemical properties

Atmospheric aerosols are complex mixtures, encompassing organic compounds, inorganic salts, and numerous other species, and thus cannot be fully represented by a simplistic, binary experimental system. The presence of inorganic salts in atmospheric aerosol have been found can affect the heterogeneous reaction kinetics of organic species, including OSs.[27, 28] Our prior study has found that the presence of ammonium sulfate (AS) in aqueous sodium dodecyl OS aerosols can significantly enhance the heterogenous $^{\bullet}$OH reaction rate by a factor of 3 as the mass ratio of AS to dodecyl sulfate increased from 0.0 to 0.77. Molecular dynamic (MD) simulations indicated that this enhancement in oxidation rate could be attributed to the preferential accumulation of ammonium ions ($NH_4^+$) at the air-water interface, along with the strong attraction between $NH_4^+$ and $^{\bullet}$OH, which facilitated the closer proximity of diffused $^{\bullet}$OH to the surface-active dodecyl sulfate ions. Despite this enhancement, reaction products remained largely consistent across the experimental aerosol compositions, suggesting a negligible impact of AS on the reaction mechanisms. Given the surface-active nature of OS species,[29] a similar reactivity enhancement is anticipated for other OSs in the presence of cations exhibiting similar surface affinity and a pronounced attraction to $^{\bullet}$OH as $NH_4^+$.

Additionally, the presence and abundance of inorganic salts can impact the ionic strength of atmospheric aerosols, thereby potentially impacting the heterogeneous and multiphase reactions. A few laboratory studies have found that high ionic strength condition can accelerate the rate of photochemical degradation of many organic compounds within aerosol and the reactive uptake of ozone on organic aerosol surface.[30, 31] This may be partly explained by that for reaction system containing an ion and a neutral reactant, the reaction rate constant can increase with the increase of ionic strength, known as the primary kinetic salt effect.[32] Taken together, the types and concentration of inorganic ions (e.g. ionic strength) within aerosol could potentially alter the overall heterogeneous kinetics of OSs while it remains an open question whether the salts alter heterogeneous reactivity chemically and thus warrant future investigations.

Furthermore, it is noteworthy that the heterogeneous $^{\bullet}OH$ oxidation process of 2-MTS can have an impact on the physicochemical properties of 2-MTS aerosols. This impact arises from both the ongoing conversion of 2-MTS to inorganic sulfate, considering that sulfur in its inorganic and organic form exhibit distinct properties[26, 33-35], and the evaporation of non-sulfated organic products during the oxidation process. For instance, compared to inorganic sulfates, 2-MTS exhibits lower hygroscopicity[26] as evidenced by measured volume growth factor ($G_f$) at 60% RH for aerosols containing ammonium sulfate ($G_f$ =~2) or 2-MTS ammonium salt ($G_f$ = 1.13). Therefore, the aerosol water uptake behaviour is expected to be enhanced upon the oxidation of 2-MTS aerosol, which may potentially impact the oxidation rate to some extent. Besides, considering the comparable pKa value of 2-MTS in its neutral form ($C_5H_{12}O_7S$, pKa = -2.37)[19] to sulfuric acid (pKa = -3.0), aerosols composed of 2-MTS anion ($C_5H_{11}O_7S^-$) and its counterion $NH_4^+$ prior to oxidation are expected to be slightly acidic. Upon the formation and dissociation of inorganic sulfates as well as the subsequent dilution resulted from enhanced water uptake, a mild decrease in aerosol pH is anticipated. The increased acidity may enhance the evaporation of low volatility organic acids products[36] and ultimately affect the aerosol mass loading. Consequently, the resulting variation in the physicochemical properties of 2-MTS aerosols may subsequently exert an influence on its heterogeneous reactivity.

Overall, we acknowledge that discussion provided above serves the purpose of gaining further qualitative insights into the potential factors driving the differences in reaction kinetics and sulfate yield observed in this experimental study compared to those in atmospheric aerosols. Further laboratory and modelling studies under more atmospherically relevant conditions are necessary to specifically address the effects of reaction conditions and aerosol properties on the heterogeneous reactivity and chemistry (e.g., sulfate formation) of 2-MTS and other isoprene-derived organosulfates.

**Table S1.** OSs formed upon the heterogeneous •OH oxidation of 2-MTS aerosols.

| No | OSs [a] | | Chemical Structure [b] | This Study [c] | Retention Time (min) | OS Yield [d] |
|---|---|---|---|---|---|---|
| | Formula ([M-H]⁻) | Measured Mass | | | | |
| 1 | $C_5H_9O_7S^-$ | 213.0074 | 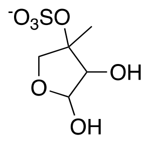 | √ | 2.54<br>2.76<br>4.88<br>5.29<br>5.81 | 0.025-0.137 [e] |
| 2 | $C_5H_7O_7S^-$ | 210.9918 | 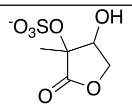 | √ | 1.51<br>1.88 | 0.0006-0.002 [f] |
| 3 | $C_5H_{11}O_8S^-$ | 231.0180 | 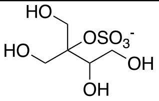 | √ | 8.61<br>8.93 | 0.004-0.013 [e] |
| 4 | $C_5H_9O_8S^-$ | 229.0024 | 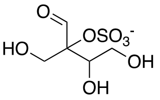 | √ | 3.66<br>4.96<br>5.73 | 0.014-0.045 [e] |
| 5 | $C_5H_7O_8S^-$ | 226.9867 | 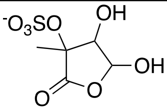 | √ | 2.31<br>2.50 | <0.001 [f] |
| 6 | $C_4H_7O_7S^-$ | 198.9918 | 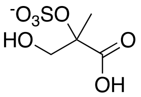 | × | - | - |
| 7 | $C_3H_7O_6S^-$ | 170.9969 | 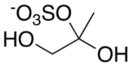 | × | - | - |
| 8 | $C_3H_5O_6S^-$ | 168.9812 | 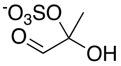 | √ | 1.21<br>11.62 | <0.001 [e] |
| 9 | $C_3H_5O_7S^-$ | 184.9763 | 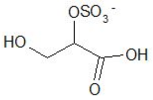 | × | - | - |
| 10 | $C_3H_8O_5S^-$ | 154.9651 | 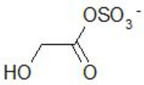 | × | - | - |
| 11 | $C_2H_3O_5S^-$ | 138.9707 | 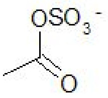 | × | - | - |

[a] These OSs were observed by HILIC/ESI-HR-QTOFMS after the heterogeneous •OH oxidation of 2-MTS aerosols in our previous filter-based study.[12]

[b] Various isomers are formed. Only one isomer was shown here for simplicity.

[c] This indicates whether the OS detected in this study. Some of our previously detected OSs were missing in this study, probably due to the low concentration of these minor OS products in diluted solutions collected by PILS.

[d] The ranges of OS yield upon oxidation were calculated using **Eqn. S1**.

[e] The quantification of this OS species for yield determination used the response factor of 2-MTS (RT = 7.17 and 8.86 min) in the HILIC/ESI-HR-QTOFMS run as an approximation.

[f] The quantification of this OS species used the response factor of methylsulfate (RT = 2.03 min) in the HILIC/ESI-HR-QTOFMS run as an approximation.

**Table S2.** The total inorganic sulfate amount measured by IC ($[SO_4^{2-}\text{-IC}]$), the inorganic sulfate formed from methylsulfate oxidation ($[SO_4^{2-}\text{-}CH_3SO_4^-]$), corrected inorganic sulfate from 2-MTS oxidation ($[SO_4^{2-}\text{-corrected}]$), and the calculated sulfate yield as a function of OH exposures.

| **$^\bullet$OH exposure (molecules cm$^{-3}$ s)** | **$[SO_4^{2-}\text{-IC}]$ (μg m$^{-3}$)** | **$[SO_4^{2-}\text{-}CH_3SO_4^-]$ (μg m$^{-3}$)** | **$[SO_4^{2-}\text{-corrected}]$ (μg m$^{-3}$) [a]** | **Sulfate Yield [b]** |
|---|---|---|---|---|
| 0 | 18.77 | 0.00 | 0.00 | 0.00 |
| $7.46 \times 10^{11}$ | 65.64 | 13.19 | 33.69 | 0.57 ± 0.14 |
| $1.09 \times 10^{12}$ | 90.94 | 20.95 | 51.22 | 0.60 ± 0.08 |
| $1.46 \times 10^{12}$ | 97.57 | 29.73 | 49.07 | 0.48 ± 0.07 |
| $1.82 \times 10^{12}$ | 119.31 | 30.35 | 70.19 | 0.60 ± 0.07 |
| $1.98 \times 10^{12}$ | 133.18 | 33.96 | 80.46 | 0.68 ± 0.07 |

[a] The corrected inorganic sulfate from 2-MTS oxidation as a function of OH exposure was calculated using **Eqn. S3**.
[b] The sulfate yield was calculated using **Eqn. S4**.

**Table S3.** Volatility of non-sulfated products formed upon the heterogeneous $^{\bullet}$OH oxidation of 2-MTS aerosols.

| **Chemical Formula** | **Chemical Structure** | **Molar weight (g mol$^{-1}$)** | **Saturation vapor pressure (Pa)** | **Effective vapor pressure, C* (μg m$^{-3}$) [d]** | **Fraction in aerosol phase [e]** |
|---|---|---|---|---|---|
| $CH_2O_2$ | 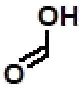 | 46 | 5888.43 [a] | $5.00\times10^{9}$ | 0.00% |
| $C_3H_6O_2$ | 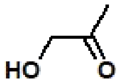 | 74 | 205.74 [b] | $6.25\times10^{6}$ | 0.00% |
| $C_2H_4O_3$ | 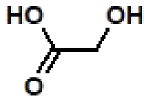 | 76 | 2.67 [c] | $1.03\times10^{5}$ | 0.29% |
| $C_4H_8O_3$ | 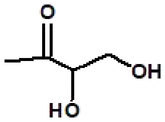 | 104 | 1.49 [b] | $6.40\times10^{6}$ | 0.47% |
| $C_4H_8O_4$ | 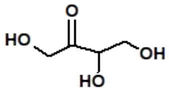 | 120 | $6.1\times10^{-3}$ [b] | $3.01\times10^{2}$ | 49.94% |

[a] This value was measured by Kahlbaum (1894).[37]

[b] The saturation vapor pressures are estimated by EVAPORATION model[38] at 293 K, which calculates the vapour pressure of an organic compound based on its molecular structure and functional groups.

[c] This value was measured by Dauber (1989).[39]

[d] C* represents the effective vapor pressure.

[e] These values refer to the mole fraction of specific organic species remaining in the aerosol phase based on equilibrium gas-aerosol partitioning calculations[40] using the measured aerosol mass loading (300-400 μg m$^{-3}$). These values were calculated by assuming a neutral solution condition and thus can serve as upper limits for aerosol-phase fraction of organic acids as their evaporation is generally enhanced with increasing acidity[36].

**References:**


1. Chen, Y., T. Dombek, J. Hand, Z. Zhang, A. Gold, A.P. Ault, K.E. Levine and J.D. Surratt, *Seasonal Contribution of Isoprene-Derived Organosulfates to Total Water-Soluble Fine Particulate Organic Sulfur in the United States.* ACS Earth Space Chem., 2021. **5**(9): p. 2419-2432.
2. Russell, G.A., *Deuterium-isotope Effects in the Autoxidation of Aralkyl Hydrocarbons. Mechanism of the Interaction of PEroxy Radicals1.* J Am. Chem. Soc., 1957. **79**(14): p. 3871-3877.
3. Bennett, J.E. and R. Summers, *Product Studies of the Mutual Termination Reactions of sec-Alkylperoxy Radicals: Evidence for Non-Cyclic Termination.* Can. J Chem., 1974. **52**(8): p. 1377-1379.
4. Bothe, E., M.N. Schuchmann, D. Schulte-Frohlinde and C.v. Sonntag, *HO2 ELIMINATION FROM α-HYDROXYALKYLPEROXYL RADICALS IN AQUEOUS SOLUTION.* Photochem. Photobiol., 1978. **28**(4-5): p. 639-643.
5. Carrasquillo, A.J., K.E. Daumit and J.H. Kroll, *Radical Reactivity in the Condensed Phase: Intermolecular versus Intramolecular Reactions of Alkoxy Radicals.* J Phys. Chem. Lett., 2015. **6**(12): p. 2388-2392.
6. George, I.J. and J.P.D. Abbatt, *Heterogeneous oxidation of atmospheric aerosol particles by gas-phase radicals.* Nature Chemistry, 2010. **2**: p. 713.
7. Kroll, J.H., C.Y. Lim, S.H. Kessler and K.R. Wilson, *Heterogeneous Oxidation of Atmospheric Organic Aerosol: Kinetics of Changes to the Amount and Oxidation State of Particle-Phase Organic Carbon.* J Phys. Chem. A, 2015. **119**(44): p. 10767-10783.
8. Kwong, K.C., M.M. Chim, J.F. Davies, K.R. Wilson and M.N. Chan, *Importance of sulfate radical anion formation and chemistry in heterogeneous OH oxidation of sodium methyl sulfate, the smallest organosulfate.* Atmos. Chem. Phys., 2018. **18**(4): p. 2809-2820.
9. Lam, H.K., K.C. Kwong, H.Y. Poon, J.F. Davies, Z. Zhang, A. Gold, J.D. Surratt and M.N. Chan, *Heterogeneous OH oxidation of isoprene-epoxydiol-derived organosulfates: kinetics, chemistry and formation of inorganic sulfate.* Atmos. Chem. Phys., 2019. **19**(4): p. 2433-2440.
10. Xu, R., Y. Ge, K.C. Kwong, H.Y. Poon, K.R. Wilson, J.Z. Yu and M.N. Chan, *Inorganic Sulfur Species Formed upon Heterogeneous OH Oxidation of Organosulfates: A Case Study of Methyl Sulfate.* ACS Earth Space Chem., 2020. **4**(11): p. 2041-2049.
11. Lai, D., Y.K. Wong, R. Xu, S. Xing, S.I.M. Ng, L. Kong, J.Z. Yu, D.D. Huang and M.N. Chan, *Significant Conversion of Organic Sulfur from Hydroxymethanesulfonate to Inorganic Sulfate and Peroxydisulfate Ions upon Heterogeneous OH Oxidation.* Environ. Sci. Technol. Lett., 2023. **10**(9): p. 773-778.
12. Chen, Y., Y. Zhang, A.T. Lambe, R. Xu, Z. Lei, N.E. Olson, Z. Zhang, T. Szalkowski, T. Cui, W. Vizuete, A. Gold, B.J. Turpin, A.P. Ault, M.N. Chan and J.D. Surratt, *Heterogeneous Hydroxyl Radical Oxidation of Isoprene-Epoxydiol-Derived Methyltetrol Sulfates: Plausible Formation Mechanisms of*

*Previously Unexplained Organosulfates in Ambient Fine Aerosols.* Environ. Sci. Technol. Lett., 2020. **7**(7): p. 460-468.

13. Kanakidou, M., J.H. Seinfeld, S.N. Pandis, I. Barnes, F.J. Dentener, M.C. Facchini, R. Van Dingenen, B. Ervens, A. Nenes, C.J. Nielsen, E. Swietlicki, J.P. Putaud, Y. Balkanski, S. Fuzzi, J. Horth, G.K. Moortgat, R. Winterhalter, C.E.L. Myhre, K. Tsigaridis, E. Vignati, E.G. Stephanou and J. Wilson, *Organic aerosol and global climate modelling: a review.* Atmos. Chem. Phys., 2005. **5**(4): p. 1053-1123.
14. Cui, T., H.S. Green, P.W. Selleck, Z. Zhang, R.E. O ' Brien, A. Gold, M. Keywood, J.H. Kroll and J.D. Surratt, *Chemical Characterization of Isoprene- and Monoterpene-Derived Secondary Organic Aerosol Tracers in Remote Marine Aerosols over a Quarter Century.* ACS Earth Space Chem.,2019. **3**(6): p. 935-946.
15. Darer, A.I., N.C. Cole-Filipiak, A.E. O'Connor and M.J. Elrod, *Formation and Stability of Atmospherically Relevant Isoprene-Derived Organosulfates and Organonitrates.* Environ. Sci. Technol., 2011. **45**(5): p. 1895-1902.
16. Hu, K.S., A.I. Darer and M.J. Elrod, *Thermodynamics and kinetics of the hydrolysis of atmospherically relevant organonitrates and organosulfates.* Atmos. Chem. Phys., 2011. **11**(16): p. 8307-8320.
17. Varelas, J.G., M.M. Vega, M.A. Upshur, F.M. Geiger and R.J. Thomson, *Synthesis Enabled Investigations into the Acidity and Stability of Atmospherically-Relevant Isoprene-Derived Organosulfates.* ACS Earth Space Chem., 2022. **6**(12): p. 3090-3100.
18. Li, Z., N. Hyttinen, M. Vainikka, O.P. Tikkasalo, S. Schobesberger and T. Yli-Juuti, *Saturation vapor pressure characterization of selected low-volatility organic compounds using a residence time chamber.* Atmos. Chem. Phys., 2023. **23**(12): p. 6863-6877.
19. Hyttinen, N., J. Elm, J. Malila, S.M. Calderón and N.L. Prisle, *Thermodynamic properties of isoprene- and monoterpene-derived organosulfates estimated with COSMOtherm.* Atmos. Chem. Phys., 2020. **20**(9): p. 5679-5696.
20. Bevington, P.R., D.K. Robinson, J.M. Blair, A.J. Mallinckrodt and S. McKay, *Data Reduction and Error Analysis for the Physical Sciences.* Comput. Phys., 1993. **7**(4): p. 415-416.
21. Che, D.L., J.D. Smith, S.R. Leone, M. Ahmed and K.R. Wilson, *Quantifying the reactive uptake of OH by organic aerosols in a continuous flow stirred tank reactor.* Phys. Chem. Chem. Phys., 2009. **11**(36): p. 7885-7895.
22. Kang, E., M.J. Root, D.W. Toohey and W.H. Brune, *Introducing the concept of Potential Aerosol Mass (PAM).* Atmos. Chem. Phys., 2007. **7**(22): p. 5727-5744.
23. Goldman, M.J., W.H. Green and J.H. Kroll, *Chemistry of Simple Organic Peroxy Radicals under Atmospheric through Combustion Conditions: Role of Temperature, Pressure, and NOx Level.* J Phys. Chem. A, 2021. **125**(48): p. 10303-10314.

24. Zhang, W., K. Issa, T. Tang and H. Zhang, *Role of Hydroperoxyl Radicals in Heterogeneous Oxidation of Oxygenated Organic Aerosols.* Environ. Sci. Technol., 2024. **58**(10): p. 4727-4736.

25. Renbaum, L.H. and G.D. Smith, *Organic nitrate formation in the radical-initiated oxidation of model aerosol particles in the presence of NOx.* Phys. Chem. Chem. Phys., 2009. **11**(36): p. 8040-8047.

26. Ohno, P.E., J. Wang, F. Mahrt, J.G. Varelas, E. Aruffo, J. Ye, Y. Qin, K.J. Kiland, A.K. Bertram, R.J. Thomson and S.T. Martin, *Gas-Particle Uptake and Hygroscopic Growth by Organosulfate Particles.* ACS Earth Space Chem., 2022. **6**(10): p. 2481-2490.

27. Xu, R., H.K. Lam, K.R. Wilson, J.F. Davies, M. Song, W. Li, Y.L.S. Tse and M.N. Chan, *Effect of inorganic-to-organic mass ratio on the heterogeneous OH reaction rates of erythritol: implications for atmospheric chemical stability of 2-methyltetrols.* Atmos. Chem. Phys., 2020. **20**(6): p. 3879-3893.

28. Madeleine Ng, S.I., K.H. Ng, P.W. Felix Yeung, R. Xu, P.-K. So, Y. Huang, J.Z. Yu, C.K.K. Choi, Y.-L. Steve Tse and M.N. Chan, *Chemical transformation of a long-chain alkyl organosulfate via heterogeneous OH oxidation: a case study of sodium dodecyl sulfate.* Environ. Sci. Atmos., 2022. **2**(5): p. 1060-1075.

29. Brüggemann, M., R. Xu, A. Tilgner, K.C. Kwong, A. Mutzel, H.Y. Poon, T. Otto, T. Schaefer, L. Poulain, M.N. Chan and H. Herrmann, *Organosulfates in Ambient Aerosol: State of Knowledge and Future Research Directions on Formation, Abundance, Fate, and Importance.* Environ. Sci. Technol., 2020. **54**(7): p. 3767-3782.

30. Mekic, M., J. Zeng, W. Zhou, G. Loisel, B. Jin, X. Li, D. Vione and S. Gligorovski, *Ionic Strength Effect on Photochemistry of Fluorene and Dimethylsulfoxide at the Air–Sea Interface: Alternative Formation Pathway of Organic Sulfur Compounds in a Marine Atmosphere.* ACS Earth Space Chem., 2020. **4**(7): p. 1029-1038.

31. Mekic, M., M. Brigante, D. Vione and S. Gligorovski, *Exploring the ionic strength effects on the photochemical degradation of pyruvic acid in atmospheric deliquescent aerosol particles.* Atmos. Environ., 2018.

32. Herrmann, H., D. Hoffmann, T. Schaefer, P. Bräuer and A. Tilgner, *Tropospheric Aqueous-Phase Free-Radical Chemistry: Radical Sources, Spectra, Reaction Kinetics and Prediction Tools.* Chem. Phys. Chem., 2010. **11**(18): p. 3796-3822.

33. Fleming, L.T., N.N. Ali, S.L. Blair, M. Roveretto, C. George and S.A. Nizkorodov, *Formation of Light-Absorbing Organosulfates during Evaporation of Secondary Organic Material Extracts in the Presence of Sulfuric Acid.* ACS Earth Space Chem., 2019: p. acsearthspacechem.9b00036-acsearthspacechem.9b00036.

34. Hansen, A.M.K., J. Hong, T. Raatikainen, K. Kristensen, A. Ylisirnio, A. Virtanen, T. Petaja, M. Glasius and N.L. Prisle, *Hygroscopic properties and cloud condensation nuclei activation of limonene-derived organosulfates and*

*their mixtures with ammonium sulfate.* Atmos. Chem. Phys., 2015. **15**(24): p. 14071-14089.

35. Zhang, Y., Y. Chen, Z. Lei, N.E. Olson, M. Riva, A.R. Koss, Z. Zhang, A. Gold, J.T. Jayne, D.R. Worsnop, T.B. Onasch, J.H. Kroll, B.J. Turpin, A.P. Ault and J.D. Surratt, *Joint Impacts of Acidity and Viscosity on the Formation of Secondary Organic Aerosol from Isoprene Epoxydiols (IEPOX) in Phase Separated Particles.* ACS Earth Space Chem., 2019. **3**(12): p. 2646-2658.
36. Wallace, B.J., M.L. Mongeau, A. Zuend and T.C. Preston, *Impact of pH on Gas-Particle Partitioning of Semi-Volatile Organics in Multicomponent Aerosol.* Environ. Sci. Technol., 2023.
37. Kahlbaum, G.W.A., *Studien über Dampfspannkraftsmessungen.* Z. Phys. Chem. (Leipzig), 1894. **13U**: p. 14 - 55.
38. Compernolle, S., K. Ceulemans and J.F. Müller, *EVAPORATION: a new vapour pressure estimation methodfor organic molecules including non-additivity and intramolecular interactions.* Atmos. Chem. Phys., 2011. **11**(18): p. 9431-9450.
39. Daubert, T.E. and R. Danner. *Physical and thermodynamic properties of pure chemicals : data compilation*. 1989.
40. Seinfeld, J.H. and J.F. Pankow, *Organic Atmospheric Particulate Material.* Annu. Rev. Phys. Chem., 2003. **54**(1): p. 121-140.
